# Autonomic Predictors of Hospitalization Due to Heart Failure Decompensation in Patients with Left Ventricular Systolic Dysfunction

**DOI:** 10.1371/journal.pone.0152372

**Published:** 2016-03-25

**Authors:** Ludmiła Daniłowicz-Szymanowicz, Justyna Suchecka, Agnieszka Niemirycz-Makurat, Katarzyna Rozwadowska, Grzegorz Raczak

**Affiliations:** Department of Cardiology and Electrotherapy, Medical University of Gdansk, Gdansk, Poland; Hospital Universitario LA FE, SPAIN

## Abstract

**Introduction:**

Autonomic nervous system balance can be significantly deteriorated during heart failure exacerbation. However, it is still unknown whether these changes are only the consequence of heart failure decompensation or can also predict development thereof. Objectives were to verify if simple, non-invasive autonomic parameters, such as baroreflex sensitivity and short-term heart rate variability can provide independent of other well-known clinical parameters information on the risk of heart failure decompensation in patients with left ventricular systolic dysfunction.

**Methods:**

In 142 stable patients with left ventricular ejection fraction ≤ 40%, baroreflex sensitivity and short-term heart rate variability, as well as other well-known clinical parameters, were analyzed. During 23 ± 9 months of follow-up 19 patients were hospitalized due to the heart failure decompensation (EVENT).

**Results:**

Pre-specified cut-off values of baroreflex sensitivity (≤2.4 ms/mmHg) and low frequency power index of heart rate variability (≤19 ms^2^) were significantly associated with the EVENTs (hazard ratio 4.43, 95% confidence interval [CI] 1.35–14.54 and 5.41, 95% CI 1.87–15.65 respectively). EVENTs were also associated with other parameters, such as left ventricular ejection fraction, NYHA class, diuretic use, renal function, brain natriuretic peptide and hemoglobin level, left atrial size, left and right ventricular heart failure signs. After adjusting baroreflex sensitivity and low frequency power index for each of the abovementioned parameters, autonomic parameters were still significant predictors of hospitalization due to the heart failure decompensation.

**Conclusion:**

Simple, noninvasive autonomic indices can be helpful in identifying individuals with increased risk of hospitalization due to the heart failure decompensation among clinically stable patients with left ventricular systolic dysfunction, even when adjusted for other well-known clinical parameters.

## Introduction

According to literature, patients with heart failure are characterized by predominance of sympathetic activity and decreased vagal tone [1–3]. Baroreflex sensitivity (BRS) and heart rate variability (HRV), reliable measures of autonomic nervous system (ANS) balance [4–6], frequently referred to as sudden cardiac death risk parameters, can be significantly deranged during acute phase of heart failure exacerbation [7, 8]. Clinical stabilization and appropriate treatment, including cardiac resynchronization or heart transplantation, can result in an improvement of autonomic balance [7–10]. However, it is still unknown if the changes in BRS and HRV values are only the consequence of heart failure decompensation or can also predict development thereof in stable patients with left ventricular (LV) systolic dysfunction.

Therefore, **objectives** of the study were to verify if BRS and HRV indices can provide independent information on the risk of hospitalization due to heart failure decompensation in stable patients with LV systolic dysfunction, and whether this information is independent of other well-established clinical parameters.

## Materials and Methods

### Patient selection

Between December 2009 and March 2012, 142 consecutive patients with chronic heart failure, presenting with typical clinical signs and symptoms of this condition [11] and LVEF≤40%, were prospectively enrolled to the study. The protocol of the study was approved by the Local Ethics Committee at the Medical University of Gdansk, and written informed consent was obtained from all participants. All patients were in sinus rhythm, optimally treated (including successful revascularization if necessary) and clinically stable for at least 3 months prior to the study. The *exclusion criteria* were: age <18 years, pacemaker rhythm including cardiac resynchronization therapy with successful resynchronization, NYHA functional class IV, permanent atrial fibrillation/flutter, permanent second- or third-degree atrioventricular block, type 1 diabetes mellitus, documented autonomic neuropathy (information based on patients records), poor general condition (concomitant terminal disease), and non-cardiologic comorbidities with potential unfavourable effect on survival.

### ANS parameters

The ANS tests were performed between 08.00 am and 1.00 pm. All patients were asked to fast for at least 4 hours and to refrain from smoking cigarettes and drinking coffee for at least 12 hours prior to the examination. The recordings were obtained in a quiet room, with the patient relaxed in the supine position with head elevated by 30°. After a 15-minute stabilization in the supine position, resting ECG (*Mingograf 720C*) and *beat-to-beat* non-invasive arterial blood pressure (*Finapres 2300*, *Ohmeda*) were recorded continuously for 10 min during spontaneous breathing. The signals were acquired with a PC workstation, processed with a dedicated software [12] and analyzed according to the protocol described elsewhere [13, 14]. The data on RR interval (resolution 1 ms) and systolic arterial pressure (SAP) were obtained automatically. BRS (ms/mmHg) was computed by spectral analysis as the average value of the transfer function modulus (Blackman-Tukey method, 0.03 Hz-bandwidth Parzen window) between SAP and RR interval time series in the low frequency (LF, 0.04–0.15 Hz) band, independently from coherence values [13]. Furthermore, routine HRV indices: LF (in ms^2^), high frequency (HF, 0.14–0.4 Hz, in ms^2^), LF to HF ratio (LF/HF), and relative spectral powers in LF and HF bands (LFnu and HFnu, expressed in normalized units) were analyzed [15]. The following time-domain HRV parameters were calculated on the basis of RR data: the standard deviation of normal-to-normal RR intervals (SDNN), the square root of the mean of squared differences between successive intervals (RMSSD), and the percentage of adjacent RR intervals differing by more than 50 ms (pNN50). Finally, mean HP (ms) value was also included in the analysis.

### Clinical parameters

Several clinical parameters that have shown a high impact predicting hospitalization for worsening of heart failure were taken into account: demographic and previous clinical history, physical examination, ECG and echocardiography parameters, laboratory blood tests, medical treatment and concomitant diseases [16]. Anemia was defined as hemoglobin <120 g/l for women and <130 g/l for men [17]. Left and right ventricular failure signs (LV and RV failure signs) were defined according to the Framingham criteria [18]. Patients were followed-up at the university outpatient clinic. The first visit took place within 3 months of enrolment; subsequently, patients were followed-up every 6 months or earlier, if clinically required. During each visit, clinical status of patient was evaluated, and any adverse events were recorded. Decision on potential implantation on an ICD as a primary preventive measure of sudden cardiac death was at the discretion of the physician in charge. The primary end-point (EVENT) of the study was an episode of hospitalization due to heart failure decompensation (including those with fatal outcome). All EVENTs were confirmed on the basis of patients’ medical documentation and/or death certificate information.

### Statistical analysis

The minimum sample size was estimated as *n* = (1.96/0.2)^2^ = 97 (the length of the 95% confidence interval (CI) would not exceed 20% [the error bound on the estimate would not exceed 10%]). In an event of potential drop-outs, the sample size was eventually set at 142 (the accuracy was improved and the error value was 8.3%). The patients’ data were censored on the date of the end-point or last follow-up. Due to the lack of normal distributions, all the variables were expressed as medians (interquartile intervals), or numbers (n) and percentages (%). The quantitative characteristics of the EVENT_(+) and EVENT_(-) groups were compared with the Mann-Whitney U-test, and the qualitative data with the chi-square test or Yates’ chi-square test. The power and direction of relationships between pairs of selected variables were analyzed on the basis of the Pearson’s coefficients of linear correlation. The accuracy of ANS indices and other parameters as potential predictors of the study end-points was determined on the basis of area (AUC) under the receiver-operating characteristic (ROC) curve. An association between analyzed parameters and the end-point was assessed using the Cox univariate and multivariate proportional hazard models, after dichotomization of the measurements according to their cut-off values that maximized the hazard ratio (HR). For this purpose, we calculated HR for progressively increasing appropriate values comprised between the 20th and 50th percentiles (to have an adequate number of patients in each subgroup) and identified the point at which HR attained its maximum. The time course of the end-point, stratified according to the aforementioned cut-off values, was estimated using the Kaplan-Meier method, and the association between compared groups was estimated by the log-rank test. All the results were considered statistically significant at p≤0.05. The statistical analysis was conducted with STATISTICA 9.0 (StatSoft, Tulsa OK, USA) package and R 2.15.2 environment.

## Results

### Clinical characteristics of the studied patients

Baseline clinical and demographic characteristics of the study group are presented in Table 1. A total of 19 patients (13%) reached the end-point during 23 ± 9 month follow-up; no episodes of malignant ventricular arrhythmia or ICD discharge were documented in those subjects before reaching the end-point of the study. Mean time elapsed between enrollment and the EVENTs was 12 ± 7 months (range 3–18 months). Patients from the EVENT_(+) group were characterized by significantly lower values of LVEF, higher left atrial (LA) size, NYHA class III, anemia, left ventricular (LV) and right ventricular (RV) failure signs, showed greater degree of functional renal impairment, higher brain natriuretic peptide (BNP) and C-reactive protein (CRP) level and used diuretics significantly more often than the EVENT_(-) individuals. 13% of patients used amiodarone: they were patients with paroxysmal atrial fibrillation in their medical history (that was not present during enrollment to the study) or patients used amiodarone due to frequent, symptomatic ventricular extrasystolies in their history.

**Table 1:** Clinical characteristics of the study group and comparison between the EVENT_(+) and EVENT_(-) groups.

	All (n = 142)	EVENT_(+) (n = 19)	EVENT_(-) (n = 123)	* *p*
Age [years]	64 (57–73)	65 (56–77)	64 (58–73)	0.29
Male, n (%)	124 (87)	17 (89)	107 (87)	1.00
BMI	27 (24–30)	27 (24–31)	27 (24–30)	0.39
CAD history, n (%)	108 (76)	14 (74)	94 (76)	0.78
Revascularization, n (%)	100 (70)	15 (79)	85 (69)	0.59
**LVEF (%)**	**30 (25–35)**	**23 (20–31)**	**30 (26–35)**	**<0.003**
**LA size (mm)**	**45 (41–49)**	**49 (46–54)**	**45 (41–48)**	**<0.001**
QRS (ms)	120 (100–140)	120 (110–150)	120 (100–135)	0.17
QRS≥120 ms, n (%)	86 (61)	14 (74)	72 (59)	0.31
**NYHA class**				**<0.001**
- NYHA I, n (%)	24 (17)	1 (5)	23 (19)	
- NYHA II, n (%)	87 (62)	7 (37)	80 (65)	
- NYHA III, n (%)	31 (22)	11 (58)	20 (16)	
**LV failure signs, n (%)**	**26 (18)**	**7 (37)**	**14 (11)**	**< 0.009**
**RV failure signs (%)**	**11 (8)**	**5 (26)**	**6 (5)**	**< 0.007**
beta-adrenolytics, n (%)	137 (96)	19 (100)	115 (94)	1.00
ACE-inhibitor or ARB, n (%)	132 (93)	17 (89)	115 (94)	0.62
spironolactone, eplerenone, n (%)	76 (54)	13 (68)	63 (51)	0.22
aspirin, n (%)	116 (82)	17 (89)	99 (80)	0.53
amiodarone, n (%)	18 (13)	3 (16)	15 (12)	0.71
statins, n (%)	120 (85)	16 (84)	104 (85)	1.00
digoxin, n (%)	9 (6)	2 (11)	7 (6)	0.36
**diuretics, n (%)**	**81 (57)**	**17 (89)**	**64 (52)**	**<0.002**
Arterial hypertension, n (%)	88 (62)	11 (58)	77 (63)	0.80
Diabetes, n (%)	39 (27)	8 (42)	31 (25)	0.17
**Renal function**				**<0.03**
-GFR>60 ml/min, n (%)	106 (75)	10 (53)	96 (78)	
-GFR 30–59 ml/min, n (%)	27 (19)	8 (42)	19 (15)	
-GFR<30 ml/min, n (%)	9 (6)	1 (5)	8 (7)	
Hypercholesterolemia, n (%)	83 (58)	11 (58)	72 (59)	1.00
**Hemoglobin (g/d)**	**142 (132–148)**	**132 (105–140)**	**144 (137–148)**	**<0.002**
**Anemia, n (%)**	**21 (16)**	**7 (39)**	**14 (11)**	**<0.009**
**BNP (pg/ml)**	**125 (85–608)**	**1364 (1001–2705)**	**106 (80–235)**	**<0.005**
**CRP (mg/l)**	**1.1 (0.5–4.5)**	**4.8 (2.0–6.63)**	**1.1 (0.5–3.1)**	**<0.01**
Natriemia (mEq/l)	139 (137–140)	138 (137–140)	139 (137–140)	0.12
-ICD, n (%)	104 (73)	16 (84)	88 (72)	0.40

Abbreviations: ACE–angiotensin converting enzyme, ARB–angiotensin receptor blockers, BMI–body mass index; BNP–brain natriuretic peptide; CAD–coronary artery disease; CABG–coronary artery bypass graft; CRP–C- reactive protein; GFR–glomerular filtration rate; ICD–implantable cardioverter-defibrillator; LA size–left atrial size; LVEF–left ventricular ejection fraction; LVEDV–left ventricular end-diastolic volume; LVESV–left ventricular end-systolic volume; LV failure signs–left ventricular heart failure signs; MI–myocardial infarction; NYHA–classification according the New York Heart Association; PCI–percutaneous coronary intervention; RV failure signs–right ventricular heart failure signs

* p value for comparison between EVENT_(+) and EVENT_(-) groups

### Autonomic parameters

A total of 36 out of the 142 (25%) recordings used to calculate the BRS and HRV indices could not be analyzed due to large number of ectopic beats or presence of artifacts. As shown in Table 2, the patients who experienced heart failure decompensation during follow-up period presented with significantly lower BRS and LF than the remaining subjects. Moreover, individuals from the EVENT_(+) group were characterized by slightly shorter mean HP and slightly lower values of LFnu, LF/HF and SDNN values than the patients from the EVENT_(-) group, but none of these differences reached a threshold of statistical significance. Futhermore, heart rate (shown in Table 2 as heart period [HP]) did not significantly differentiate the two examined groups of patients.

**Table 2:** BRS and HRV parameters of patients from the EVENT_(+) and EVENT_(-) groups.

	All (n = 106)	EVENT_(+) (n-13)	EVENT _(-) (n = 93)	*p
Mean HP (ms)	1045 (958–1166)	1012 (924–1164)	1052 (959–1165)	0.27
SDNN (ms)	24.85 (15.45–36.55)	16.34 (10.26–33.38)	25.25 (17.73–37.02)	0.12
RMSSD (ms)	17.45 (9.85–31.33)	15.05 (7.21–31.13)	17.65 (10.07–31.185)	0.29
pNN50 (%)	0.55 (0–8.80)	0.39 (0–8.35)	0.55 (0–8.88)	0.49
**LF (ms**^**2**^**)**	**73.30 (22.88–237.00)**	**16.05 (5.11–255.50)**	**80.65 (32.60–230.80)**	**<0.04**
HF (ms^2^)	86.00 (28.75–253.55)	74.15 (14.40–292.22)	57.40 (32.60–75.48)	0.24
LFnu	44.40 (24.63–67.43)	36.40 (17.28–58.65)	47.25 (25.75–68.10)	0.15
HFnu	57.40 (32.60–75.48)	63.60 (41.35–82.73)	53.95 (31.90–75.33)	0.17
LF/HF	0.80 (0,33–2.07)	0.57 (0.21–1.42)	0.90 (0.35–2.14)	0.15
**BRS(ms/mmHg)**	**4.85 (2.43–7.38)**	**2.33 (1.54–4.81)**	**4.95 (2.61–7.391)**	**<0.03**

Abbreviations: HP–heart period; SDNN–standard deviation of the average R-R intervals of the sinus rhythm; RMSSD–square root of the mean squared difference of successive R-R intervals; pNN50 –proportion of successive R-R intervals that differ by more than 50 ms; LF–spectral power in low-frequency range (0.04–0.15 Hz); HF–spectral power in high-frequency range (0.15–0.4 Hz); LFnu–relative spectral power in LF range, expressed in normalized units; HFnu–*r*elative spectral power in HF range, expressed in normalized units; LF/HF–LF to HF ratio; BRS–baroreflex sensitivity

* p value for comparison between EVENT_(+) and EVENT_(-) groups.

We did not find significant linear correlations between BRS and LF values (i.e. the parameters that differed significantly between the EVENT_(+) and EVENT_(-) groups) and LVEF (*R* = 0.02, *P* = 0.81 and *R* = 0.07, *P* = 0.49 for EVENT_(+) and EVENT_(-), respectively). In turn, a significant linear correlation between BRS and LF values was observed (*R* = 0.56, *P* < 0.001); on subgroup analysis this correlation turned out to be particularly strong in the case of the EVENT_(+) patients (*R* = 0.95, *P* < 0.001 vs. *R* = 0.59, *P* < 0.001 in the EVENT_(-) group).

### Predictors of hospitalization due to heart failure

Analysis of the ROC curve identified LVEF and BNP as the most accurate predictor of hospitalization due to heart failure among all the studied continuous variables (AUC 71.2% [CI 57.2–85.2%] for LVEF and AUC 89.8% [CI 79.5–100.0%] for BNP). BRS (AUC 66.5% [CI 45.8–87.2%]) and LF (AUC 64.0% [CI 43.8–84.2%]) were characterized by lower discriminatory powers.

**Fig 1:**
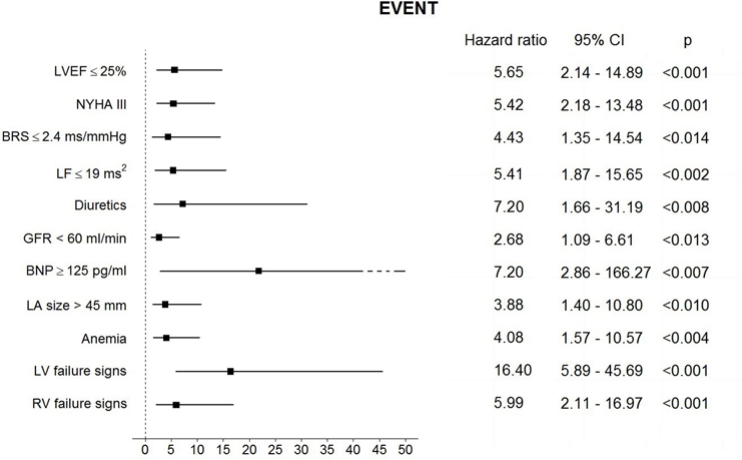
Results of Cox proportional hazard regression analysis for the pre-specified cut-off values of analyzed parameters as predictors of the EVENT during follow-up period. The central estimate and 95% confidence interval for the hazard ratio is shown.

The cut-off values optimally identifying patients at increased risk of the EVENT were: BRS ≤ 2.4 ms/mmHg, LF ≤ 19 ms^2^, LVEF ≤ 25%, glomerular filtration rate (GFR) less than 60 ml/min, LA size > 45 mm and BNP ≥ 125 pg/ml. All these values, as well as NYHA class III, anemia, LV- and RV failure signs and diuretic use, turned out to be significantly associated with the incidence of the end-point on univariate Cox analysis (Fig 1). Natriemia and CRP level was not significantly associated with the EVENTs on univariate Cox analysis.

**Fig 2:**
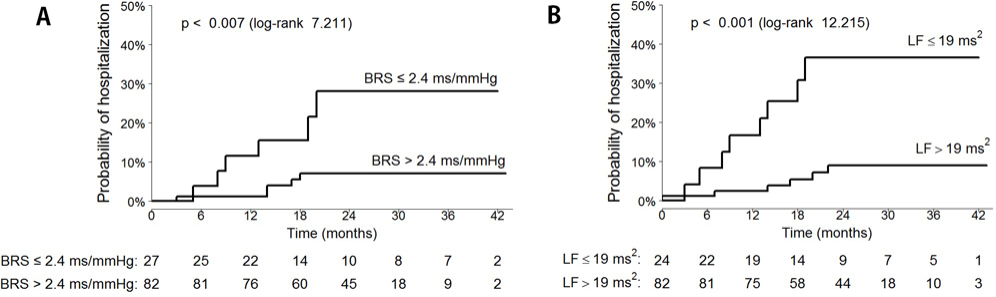
Kaplan-Meier curves illustrating probability of the EVENT depending on pre-specified cut-off values of analyzed autonomic parameters, during follow-up period. Estimated EVENT rates (95% CI) at 12 and 24 months are: (**A)** 11.5% (0.0–23.0) and 28.1% (5.0–45.6), respectively, for BRS≤2.4 ms/mmHg, and 1.2% (0.0–3.6) and 7.1% (0.9–12.9), respectively, for BRS>2.4 ms/mmHg; **(B)** 16.7% (0.3–30.3) and 36.5% (12.1–54.2), respectively, for LF≤19 ms^2^, and 2.4% (0.0–5.7) and 9.0% (1.7–15.8), respectively, for LF>19 ms^2^.

Accuracy of the abovementioned cut-off values of BRS, LF and other estimated parameters in predicting the risk of the EVENT is presented in Table 3. Fig 2 illustrates Kaplan-Meier curves the probability of reaching the end-point depending on BRS and LF values.

**Table 3:** Prognostic accuracy of the analyzed ANS parameters and composite measures (for the pre-specified cut-off values) as predictors of the EVENT.

		Characteristics (95% CI)		Predictive Value (95% CI)	
Parameters	AUC (%)	Sensitivity (%)	Specifity (%)	Positive (%)	Negative (%)
LVEF≤25%	72.02	68.42 (46.00–84.64)	75.61(67.32–82.35)	30.23 (18.60–45.11)	93.94 (87.40–97.19)
NYHA III	70.82	57.89 (36.28–76.86)	83.74(76.22–89.22)	35.48 (21.12–53.05)	92.79 (86.42–96.30)
BRS ≤ 2.4 ms/mmHg	66.14	36.36 (15.17–64.62)	95.93(89.97–98.40)	50.00 (21.52–78.48)	93.07 (86.38–96.60)
LF ≤ 19 ms^2^	65.68	35.71 (16.34–61.24)	95.65(89.35–98.30)	55.56 (26.67–81.12)	90.72 (83.30–95.04)
Diuretics	68.72	89.47 (68.61–97.06)	47.97(39.33–56.72)	20.99 (13.54–31.07)	96.72 (88.81–99.10)
GFR < 60 ml/min	62.71	47.37 (27.33–68.29)	78.05(69.95–84.45)	25.00 (13.75–41.07)	90.57 (83.50–94.79)
BNP ≥ 125 pg/ml	77.52	93.75 (71.67–99.68)	61.29(48.85–72.42)	38.46 (24.89–54.10)	97.44 (86.82–99.87)
LA size > 45 mm	66.93	73.68 (51.21–88.19)	60.18(50.96–68.72)	23.73 (14.69–35.97)	93.15 (84.95–97.04)
Anemia	63.46	38.89 (20.31–61.38)	88.03(80.91–92.74)	33.33 (17.19–54.63)	90.35 (83.54–94.53)
LV failure signs	81.96	73.68 (51.21–88.19)	90.24(83.72–94.33)	53.85 (35.46–71.24)	95.69 (90.31–98.15)
RV failure signs	60.71	26.32 (11.81–48.79)	95.12(89.77–97.75)	45.45 (21.27–71.99)	89.31 (82.86–93.53)
LVEF ≤ 25% + BRS ≤ 2.4 ms/mmHg	66.14	36.36 (15.17–64.62)	95.92 (89.97–98.40)	50.00 (21.52–78.48)	93.07 (86.30–96.60)
LVEF ≤ 25% + LF ≤ 19 ms^2^	65.68	35.71 (16.34–61.24)	95.65 (89.35–98.30)	55.56 (26.67–81.12)	90.72 (83.30–95.04)
NYHA III + BRS ≤ 2.4 ms/mmHg	62.11	27.27 (9.75–56.56)	96.94 (91.38–98.95)	50.00 (18.76–81.24)	92.23 (85.42–96.01)
NYHA III + LF ≤ 19 ms^2^	63.20	28.57 (11.72–54.65)	97.83 (92.42–99.40)	66.67 (30.00–90.32)	90.00 (82.56–94.48)
Diuretics + BRS ≤ 2.4 ms/mmHg	72.17	54.55 (28.01–78.73)	89.80 (82.23–94.36)	37.50 (18.48–61.36)	94.62 (88.03–97.68)
Diuretics + LF ≤ 19 ms^2^	57.14	14.29 (4.01–39.94)	100.00 (95.99–100.00)	39.58 (19.68–62.37)	88.46 (80.91–93.28)
GFR < 60 ml/min + BRS≤2.4 ms/mmHg	56.03	18.18 (5.14–47.70)	93.88 (87.28–97.16)	25.00 (7.15–59.07)	91.09 (83.93–95.24)
BNP ≥ 125 pg/ml + BRS ≤ 2.4 ms/mmHg	69.90	50.00 (23.66–76.34)	89.80 (78.24–95.56)	50.00 (23.66–76.34)	89.80 (78.24–95.56)
BNP ≥ 125 pg/ml + LF ≤ 19 ms^2^	58.33	16.67 (4.70–44.80)	100.00 (92.29–100.00)	51.00 (28.65–76.35)	82.14 (70.16–90.00)
LA size > 45 mm + BRS ≤ 2.4 ms/mmHg	55.76	18.18 (5.14–47.70)	93.33 (86.21–96.91)	25.00 (7.15–59.07)	90.32 (82.62–94.82)
LA size > 45 mm + LF ≤ 19 ms^2^	61.34	28.57 (11.72–54.65)	94.12 (86.96–97.46)	44.44 (18.88–73.33)	88.89 (80.74–93.85)
Anemia + BRS ≤ 2.4 ms/mmHg	56.96	18.18 (5.14–47.70)	95.74 (89.56–98.33)	33.33 (9.68–70.00)	90.91 (83.62–95.14)
Anemia + LF ≤ 19 ms^2^	53.57	7.14 (0.37–31.47)	100.00 (95.86–100.00)	41.15 (26.79–79.9)	87.25 (79.41–92.40)
LV failure signs + BRS ≤ 2.4 ms/mmHg	66.65	36.36 (15.17–64.62)	96.94 (91.38–98.95)	57.14 (25.05–84.18)	93.14 (86.51–96.64)
LV failure signs + LF ≤ 19 ms^2^	66.65	36.36 (15.17–64.62)	96.94 (91.38–98.95)	57.14 (25.05–84.18)	93.14 (86.51–96.64)
RV failure signs + BRS ≤ 2.4 ms/mmHg	57.56	18.18 (5.14–47.70)	96.94 (91.38–98.95)	40.00 (11.76–76.93)	91.35 (84.37–95.38)
RV failure signs + LF ≤ 19 ms^2^	53.57	7.14 (0.37–31.47)	100.00 (95.99–100.00)	42.13 (21.78–77.95)	87.62 (79.96–92.62)

Abbreviations: AUC–area under the receiver-operating characteristic (ROC) curve, BRS–baroreflex sensitivity; BNP–brain natriuretic peptide; CI–confidence interval; GFR–glomerular filtration rate; LA size–left atrial size; LF–spectral power in low-frequency range (0.04–0.15 Hz); LVEF–left ventricular ejection fraction; LV failure signs–left ventricular heart failure signs; NYHA–classification according the New York Heart Association; RV failure signs–right ventricular heart failure signs

### Composite predictors of hospitalization due to heart failure

As a total of 19 EVENTs were documented among 142 patients, the maximum number of predictors that could be used in a multivariate model without the risk of its over-fitting was 2. Therefore, we tested separately combinations of LVEF, NYHA class, diuretic use, GFR, BNP, LA size, anemia, LV and RV failure signs, with the cut-off value of one of the analyzed autonomic parameters (Table 4). Either BRS or LF (as well as all mentioned above parameters) proved to be significant predictors of the end-point in each of these models.

**Table 4:** Results of multivariate Cox proportional hazard regression analysis for the pre-specified cut-off values of analyzed parameters as predictors of the EVENT during follow-up period.

	Hazard ratio (95% CI)	p
LVEF-adjusted HR for BRS ≤2.4 ms/mmHg	4.17 (1.27–13.7)	<0.02
LVEF-adjusted HR for LF≤19 ms^2^	5.35 (1.83–15.58)	<0.002
NYHA III—adjusted HR for BRS ≤2.4 ms/mmHg	3.84 (1.16–12.73)	<0.03
NYHA III—adjusted HR for LF≤19 ms^2^	5.28 (1.8–15.45)	<0.002
Diuretics—adjusted HR for BRS ≤ 2.4 ms/mmHg	4.08 (1.24–13.43)	<0.021
Diuretics—adjusted HR for LF ≤ 19 ms^2^	4.53 (1.55–13.21)	<0.006
GFR < 60—adjusted HR for BRS ≤ 2.4 ms/mmHg	4.33 (1.34–14.5)	<0.017
GFR < 60—adjusted HR for LF ≤ 19 ms^2^	4.65 (1.5–14.4)	<0.008
BNP—adjusted HR for BRS ≤ 2.4 ms/mmHg	3.17 (1.07–12.7)	<0.036
BNP—adjusted HR for LF ≤ 19 ms^2^	4.39 (1.31–14.73)	<0.017
LA size—adjusted HR for BRS ≤ 2.4 ms/mmHg	4.8 (1.45–15.84)	<0.01
LA size—adjusted HR for LF ≤ 19 ms^2^	5.28 (1.82–15.31)	<0.002
Anemia—adjusted HR for BRS ≤2.4 ms/mmHg	4.32 (1.29–14.4)	<0.017
Anemia—adjusted HR for LF≤19 ms^2^	4.72 (1.59–14.03)	<0.005
LV failure signs—adjusted HR for BRS ≤2.4 ms/mmHg	3.19 (1.09–13.0)	<0.035
LV failure signs—adjusted HR for LF≤19 ms^2^	5.43 (1.81–16.33)	<0.003
RV failure signs—adjusted HR for BRS ≤2.4 ms/mmHg	3.54 (1.03–12.17)	<0.035
RV failure signs—adjusted HR for LF≤19 ms^2^	4.13 (1.37–12.51)	<0.012

Abbreviations: BRS–baroreflex sensitivity; BNP–brain natriuretic peptide; CI–confidence interval; GFR–glomerular filtration rate; LA size–left atrial size; LF–spectral power in low-frequency range (0.04–0.15 Hz); LVEF–left ventricular ejection fraction; LV failure signs–left ventricular heart failure signs; NYHA–classification according the New York Heart Association; RV failure signs–right ventricular heart failure signs

**Fig 3:**
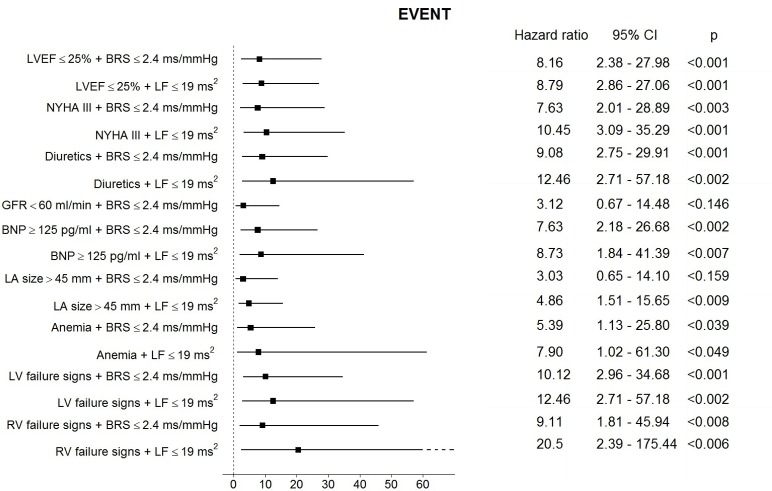
Results of Cox proportional hazard regression analysis for the pre-specified cut-off values for combinations of analyzed parameters as predictors of the EVENT during follow-up period. The central estimate and 95% confidence interval for the hazard ratio is shown.

The data on prognostic accuracy of composite measures is presented in Table 3. The results of Cox analysis that identified various combinations of the analyzed autonomic parameters as significant composite predictors of the EVENT are demonstrated on Fig 3. Combination of GFR < 60 ml/min and LF ≤ 19 ms^2^ was not present due to the lack EVENts in this group.

## Discussion

The observation that decreased BRS and LF may predict hospitalization due to heart failure decompensation in clinically stable patients with LV systolic dysfunction, even after adjusting for other well-known clinical parameters (such as LVEF, NYHA class, impaired renal function, diuretics using, BNP level, anemia, LA size, LV and RV failure signs), is the principal finding of our study. The cut-off values determined in this study (BRS ≤2.4 ms/mmHg and LF ≤19 ms^2^) accurately identified the patients who were at increased risk of heart failure decompensation during nearly two-year follow-up. A significant association between mentioned above clinical markers (LVEF, NYHA class, impaired renal function, diuretics using, BNP level, anemia, LA size, LV and RV failure signs) and the risk for heart failure decompensation was also confirmed.

### Prognostic role of autonomic nervous system parameters

To date, the prognostic value of BRS and HRV testing was unambiguously confirmed solely with regards to malignant ventricular arrhythmias, sudden cardiac death and cardiac mortality [14, 19–23]. Although a number of authors documented alterations of these parameters in individuals with heart failure [1–3, 7, 23, 24], the data on the prognostic value of BRS and HRV as potential predictors of heart failure decompensation in clinically stable patients with LV systolic dysfunction, treated according to current guidelines, are sparse. One study showed that baseline HRV may be useful in identifying patients being at increased risk of hospitalization due to late-onset heart failure after acute myocardial infarction [25]. Another study of more than 4 thousand healthy volunteers, conducted by Shan et al., showed that significantly decreased values of short-term RMSSD and SDNN constitute risk factors for development of symptomatic heart failure [26]. Nolan et al. revealed that a decrease in SDNN, documented on 24-hour Holter monitoring, is a predictor of death due to progressive heart failure in patients with chronic form of this condition [27].

Our findings are consistent with the abovementioned data, showing that both BRS and LF can predict hospitalization due to heart failure decompensation. However, contrary to previous studies, our group was comprised of stable patients with LV systolic dysfunction, treated according to current guidelines, i.e. receiving an array of beta-blockers among other therapeutic agents. The novelty of the present study can be found in the demonstration that simple autonomic indices, obtained noninvasively from short-term SAP and ECG signals, are independent predictors of hospitalization due to heart failure decompensation, even after adjusting for other, well-established clinical parameters, in patients with left ventricular dysfunction.

### Possible pathophysiological mechanism

Heart failure can be considered a state of autonomic imbalance, namely a generalized sympathetic activation combined with a relative decrease in parasympathetic tone [28, 29]. The mechanism through which autonomic markers predicted the risk of heart failure decompensation in our patients is not clear. Decreased cardiac output is postulated to enhance the sympathetic tone and cause a resultant decrease in the parasympathetic stimulation; therefore, changes in LVEF to alterations of ANS parameters [30]. However, a relatively weak association between BRS or LF with LVEF, both described in literature [31] and documented in the present study, points to likely involvement of other factors in this process. For example, according to Huikuri et al., a decrease in BRS and HRV parameters observed in heart failure patients may reflect potential neurohormonal activation, associated with LV remodeling and progression of HF [2]. Our hereby presented findings, documenting that decreased BRS and short-term LF indices can represent important physio-pathological pathways of heart failure decompensation, but this aspect is speculative and not supported by the results of the present study. These parameters as powerful predictors after adjusted for other well-known clinical parameters (LVEF, NYHA class, impaired renal function, diuretics using, BNP level, anemia, LA size, LV and RV failure signs), justify further research on potential mechanisms linking ANS dysfunction to heart failure decompensation.

We are well aware of potential ***limitations*** of our study. This was a small, single-center study, and thus its results need to be confirmed on a larger group of patients. Additionally, ANS parameters (BRS and HRV) could not be analyzed in 25% of included patients, which narrows even more the group of patients. This, unfortunately, is a known problem of ANS tests described in published documents in this field. Furthermore, we did not analyze neither invasive hemodynamic parameters or dynamics of the ANS indices before reaching the end-point, nor the ANS parameters at the moment of patients’ hospitalization due to the heart failure exacerbation. All these data would be useful for detailed understanding of pathophysiological mechanisms underlying the hereby reported phenomena. Unfortunately, so far, it has been difficult to unequivocally determine the pathophysiological mechanism linking ANS parameters and the factors leading to the decompensated phase of heart failure and hospital admission of patients.

## Conclusions

The results of our study suggest that simple noninvasively obtained parameters of ANS activity, such as BRS and short-term LF, can be helpful in identifying individuals at increased risk of hospitalization due to heart failure decompensation among clinically stable patients with LV systolic dysfunction, even when adjusted for other simple clinical parameters, such as LVEF and NYHA class. These findings substantiate further research during which patients with systolic LV dysfunction would be subjected to repeated BRS and HRV analyzes in order to study the dynamics of changes in ANS activity and to identify short- and long-term predictors of heart failure decompensation.
